# In Vivo Imaging of Enteric Neurogenesis in the Deep Tissue of Mouse Small Intestine

**DOI:** 10.1371/journal.pone.0054814

**Published:** 2013-01-31

**Authors:** Kei Goto, Go Kato, Isao Kawahara, Yi Luo, Koji Obata, Hiromi Misawa, Tatsuya Ishikawa, Hiroki Kuniyasu, Junich Nabekura, Miyako Takaki

**Affiliations:** 1 Department of Physiology II, Nara Medical University, School of Medicine, Kashihara, Nara, Japan; 2 Department of Molecular Pathology, Nara Medical University, School of Medicine, Kashihara, Nara, Japan; 3 Division of Homeostatic Development, Department of Developmental Physiology, National Institute for Physiological Sciences, Okazaki, Aichi, Japan; University of California, Los Angeles, United States of America

## Abstract

One of the challenges of using imaging techniques as a tool to study cellular physiology has been the inability to resolve structures that are not located near the surface of the preparation. Nonlinear optical microscopy, in particular two photon-excited fluorescence microscopy (2PM), has overcome this limitation, providing deeper optical penetration (several hundred µm) in ex vivo and in vivo preparations. We have used this approach in the gut to achieve the first in vivo imaging of enteric neurons and nerve fibers in the mucosa, submucosa, submucosal and myenteric plexuses, and circular and longitudinal muscles of the small intestine in H-line: Thy1 promoter GFP mice. Moreover, we obtained clear three-dimensional imaging of enteric neurons that were newly generated after gut transection and reanastomosis. Neurogenesis was promoted by oral application of the 5-HT_4_-receptor agonist, mosapride citrate (MOS). The number of newly generated neurons observed in mice treated with MOS for one week was 421±89 per 864,900 µm^2^ (n = 5), which was significantly greater than that observed in preparations treated with MOS plus an antagonist (113±76 per 864,900 µm^2^) or in 4 week vehicle controls (100±34 per 864,900 µm^2^) (n = 4 both). Most neurons were located within 100 µm of the surface. These results confirm that activation of enteric neural 5-HT_4_-receptor by MOS promotes formation of new enteric neurons. We conclude that in vivo 2PM imaging made it possible to perform high-resolution deep imaging of the living mouse whole gut and reveal formation of new enteric neurons promoted by 5-HT_4_-receptor activation.

## Introduction

Activation of enteric neural 5-HT_4_-receptors by mosapride citrate (MOS) promotes the reconstruction of an enteric neural circuit injured after surgery, leading to the recovery of the ‘defecation reflex’ [1], [2] in the distal gut of guinea pigs [3]. This neural plasticity involves neural stem cells [3]. Recently, we also revealed that MOS enhances neural network formation in gut-like organs differentiated from mouse embryonic stem cells [4]. Other 5-HT_4_ receptor agonists also increase neuronal numbers and length of neurites in enteric neurons developing in vitro from immunoselected neural crest-derived precursors [5]. 5-HT_4_ receptor-mediated neuroprotection and neurogenesis has also been demonstrated in the enteric nervous system of adult mice [6]. We therefore explored the ability of MOS to promote the generation of new enteric neurons at resected sites of the mouse small intestine *in vivo*. The new neurons are typically located in regions of granulation tissue, which is new connective tissue formed by growth of fibroblasts and blood capillaries into injured tissue after transection and reanastomosis of the gut.

Unfortunately, it is impossible for traditional fluorescence microscopy including confocal microscopy to perform high-resolution deep imaging of the 300–400 µm thick granulation tissue that is formed during the tissue repairing process at the anastomotic site after transection of the gut. Even in in vitro whole mount preparations, in which the mucosal, submucosal and circular muscle layers were removed, imaging of newly formed neurons and axons is severely limited. Nonlinear optical microscopy, in particular two photon-excited fluorescence microscopy, offers a means to overcome this limitation by providing enhanced optical penetration. Two-photon microscopy (2PM) allows cellular imaging several hundred microns deep in various organs of living animals and ex vivo specimens [7].

In the present study, we employed 2PM to obtain 3-dimensional reconstructions of impaired enteric neural circuits within the thick granulation tissue in the ileum of Thy1-GFP mice [8], in which the GFP is expressed in the cytoplasm of enteric neurons. Although in vivo imaging of the muscularis propria and myenteric neurons with probe-based confocal laser endomicroscopy in porcine models has been recently reported [9], we obtained the first ever (deleted) clear three-dimensional imaging of newly generated enteric neurons within the thick granulation tissue at the anastomosis, indicating that 2PM allows enteric neural imaging several hundred microns deep in the gut of the living mouse. The most critical challenge was to suppress movement artifacts to allow for microscopy in the living gut. In addition, we tested whether activation of enteric neural 5-HT_4_-receptors by MOS promotes reconstruction of an enteric neural circuit injured after the surgery as has been demonstrated in the lower gut [3].

## Materials and Methods

### Description and Preparation of Transgenic Mice Used for Imaging

All relevant experimental protocols were approved by the Ethics Review Committee for Animal Experimentation of the National Institutes for Physiological Sciences (permission number: 11A114). We used a transgenic mouse, based on the C57BL/6 strain, with sparse expression of cytoplasmic GFP in thalamic and cortical pyramidal neurons (Thy1 promoter GFP mouse, H-line) [8]. In preliminary studies, we confirmed expression of cytoplasmic GFP in enteric neurons. Transgenic mice, at 8–12 weeks after birth, were anesthetized with an intraperitoneal injection of Nembutal (50 mg kg^−1^) and the abdomen was opened by a lower midline laparotomy. This approach spared vascular perfusion and maintained extrinsic inputs from the mesenteric nerves. The ileum was transected 5–6 cm from ileo-cecal sphincter and an end-to-end one-layer anastomosis was performed. Body temperature was maintained at 36–37°C using a heating pad. After recovery from the surgical procedure, mouse daily drank 0.1% DMSO vehicle solution (n = 5), MOS (100 µM) in vehicle (n = 6), or a selective 5-HT_4_-blocker for oral application, SB-207266 (SB: 10–50 µM) [10] plus MOS (100 µM) in vehicle (n = 4) for 1 week (with the beginning two days fasting and the ending five days feeding ad libitum). In addition, vehicle controls were maintained for 4 weeks (n = 4) with the beginning two days fasting and the ending twenty-six days feeding.

### In vivo Two-photon Microscopy

**Figure 1:**
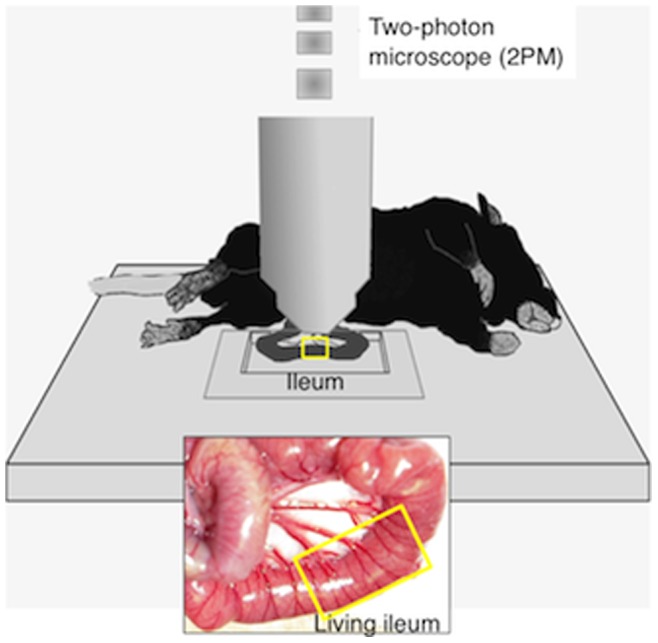
Depiction of the experimental approach for *in vivo* imaging of the intestine.

Seven days after the surgery, the mice were anesthetized with Nembutal (50 mg kg^−1^) for in vivo microscopy. Body temperature was maintained at 36–37°C using a disposable pocket body warmer. Additional Nembutal was administered as needed. The depth of anesthesia was assessed by monitoring respiration rate and vibrissae movements. The abdomen was opened by a lower midline laparotomy and the surgical site of the ileum was fixed into the chamber for 2PM without disturbance of blood supply (**Figure 1**). To suppress ileal motility for microscopy, the preparation was pinned in place and papaverine (1 mM; 0.1–0.2 ml) was injected intraluminally. Images were obtained at the rate of 2.711 sec frame^−1^. GFP-labeled structures were imaged by a 2PM customized for in vivo imaging (FV1000-MPE, Olympus, Tokyo) using a water-immersion objective lens (40X, NA0.8, Olympus) at zoom of 1.0. A Ti-sapphire laser (MaiTai Hp, Spectral Physics, Mountain View, CA, USA) was tuned to the excitation wavelength for GFP (950 nm). The z-stack images consisted of 100–300 optical sections and were taken 1 µm apart (within 400 µm of the serosal surface). The photomultiplier tube setting and excitation power (∼50 mW) remained constant during imaging. Under these conditions, the neurons seem to be still healthy after imaging (personal communication from Dr. Go Kato).

### Immunohistochemical Imaging by Confocal Microscope

Following in vivo imaging, the animals were euthanized by administration of excessive dose of Nembutal, and whole mount preparations of the anastomosed regions of ileum were fixed in 4% paraformaldehyde (4°C, overnight) or 99.5% acetone (4°C, 1 hr) to detect neurofilament (NF). Thereafter, the mucosa and submucosa and granulation tissue were carefully removed, and following a 30 min wash in PBS (0.01–0.1 mol L^−1^, pH 7.4) the preparations were incubated for 3–12 hrs at 4°C in 10% normal donkey serum in PBS containing 0.3% (v/v) Triton-X 100 (PBS-TX) to enhance penetration of antibodies. The preparations were then incubated for two days at 4°C with a rabbit polyclonal antiserum cocktail to label NF (clone 2F11, reacting with 70, 160 and 200 kDa proteins, 0.5 µg/ml; DAKO). NF immunoreactivity was detected using an Alexa Fluor 594-conjugated secondary antibody (Invitrogen Inc., Carisbad, CA). Tissues were examined with an OLYMPUS FV1000 (Tokyo, Japan) confocal microscope. Confocal images were constructed as digital composites of Z-series scans of 10–15 optical sections through a depth of 10–20 µm or 100–150 µm. Final images were produced with the FV10-ASW software application [Ver1.7] (OLYMPUS).

### Immunohistochemistry of Sectioned Preparations

The rectum including an anastomotic site was fixed with 4% paraformaldehyde at 4°C, and embedded in paraffin. Consecutive 4 µm sections were cut from each block. Immunostaining was performed by treatment with pepsin (DAKO Corp., Carpinteria, CA, USA) for 20 min at room temperature for NF, DLX2, GFP and GFAP. After endogenous peroxidase blockade with 3% H_2_O_2_-methanol for 15 min, specimens were rinsed with PBS and incubated with a primary antibody diluted with Washing Solution (BioGenex, San Ramon, CA, USA) at room temperature for 2 hours. The specimens were rinsed with PBS and incubated at room temperature for 1 hour with secondary antibody conjugated to peroxidase diluted at 0.5 µg mL^−1^ (Medical & Biotechnological Laboratories Co., Ltd., Nagoya, Japan). The sections were then rinsed with PBS and color-developed by diaminobenzidine (DAB) solution (DAKO) and counterstained with Meyer’s hematoxylin (Sigma Chemical Co., St. Louis, MO, USA). Antibodies used in primary reaction and the working concentrations were as followed: anti-NF (clone 2F11, reacting with 70, 160, and 200 kDa proteins, 0.5 µg mL^−1^, DAKO), anti-distal less homeobox 2 (DLX2)(cat. ab18188, 0.5 µg mL^−1^, Abcam Co, Tokyo, Japan) as an enteric neural stem cell marker, anti-green fluorescent protein (GFP)(0.5 µg mL^−1^, Rockland Immunochemicals Inc., Gilbertsville, PA) and glial fibrillary acidic protein (GFAP)(0.5 µg mL^−1^, DAKO Corp, Carpintaria, CA) as an enteric glia cell marker.

### Detection of Regenerated Enteric Neurons

To identify neuronal cell proliferation, 5-bromo-2-deoxyuridine, BrdU (1 mg mL^−1^ solution; Sigma or NACALAI TESQUE, INC, Kyoto, Japan) was added to the drinking water containing MOS (100 µM) for 1–2 weeks for 6 animals. After rinsing in PBS, the specimens were pretreated with sodium chloride sodium citrate solution for 2 hrs at 65°C, followed by partial denaturation of double-stranded DNA with 2 mol L^−1^ HCl for 30 min at 37°C. To reveal BrdU, the sections were incubated with a rat monoclonal antibody raised against BrdU (Abcam Inc.) overnight at 4°C. The specimens were rinsed in 0.1 mol L^−1^ TE (pH 7.8) followed by routine immunohistochemistry.

### Statistics

Multiple comparisons were performed by one-way analysis of variance (ANOVA) with post-hoc Bonferroni’s test. A value of p<0.05 was considered statistically significant. All data are expressed as the mean ± S.D.

## Results

**Figure 2:**
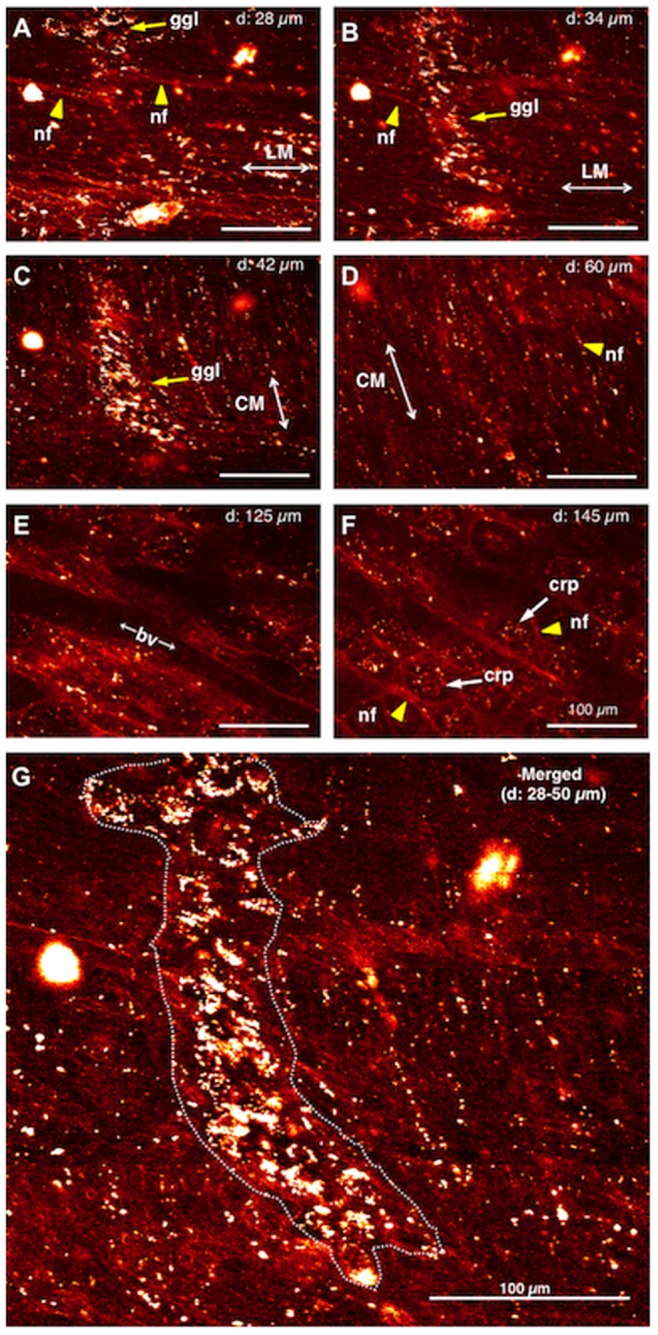
In vivo imaging of enteric neurons in the terminal ileum of an intact Thy1-GFP mouse. *A.* 28 µm deep to the serosal surface. ***B***. 34 µm deep to the serosal surface. ***C***. 42 µm deep to the serosal surface. ***D***. 60 µm deep to the serosal surface. ***E.*** 125 µm deep to the serosal surface. ***F.*** 145 µm deep to the serosal surface. ***G.*** Merge of 28–50 µm deep images into a single image. Yellow arrows indicate ganglion (ggl) in ***A–C***, and yellow arrowheads indicate nerve fibers in ***A, B, D*** and ***F***, respectively. LM: longitudinal muscle. CM: circular muscle. bv: blood vessel. crp: crypt. Cal bar, 100 µm.

In the current study, we obtained the first in vivo images of enteric neurons and nerve fibers in the mucosa, submucosa, submucosal and myenteric plexuses, and circular and longitudinal muscles of the terminal ileum (**Figure 2**). We initially confirmed that enteric neurons could be imaged in vivo in the terminal ileum of an intact Thy1-GFP mouse (**Figure 2**). The normal mouse gut was rather thin, with a maximal thickness at myenteric ganglion level of 50 µm. Although images stacked with a Z-axis depth up to 50 µm showed low signal/background fluorescence intensity ratio, each image at depth of 28 µm–42 µm from serosa showed a single myenteric ganglion and longitudinal & circular muscle layers (**Figure 2** ***A–C***). Thicker images at depth of 60, 125 and 145 µm also showed nerve fibers in circular muscle layers, blood vessels and nerve fibers around crypts (Figure 2D–F), respectively. Living cells in a given ganglion were clearly observed in merged images at depth of 28 µm–50 µm (**Figure 2** ***G***).

**Figure 3:**
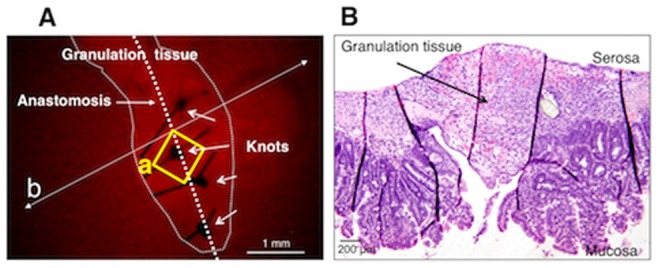
A stereomicroscopic image including the observed site shown in **Figure 4**. ***A.*** The thick granulation tissue at the anastomotic region in a mouse that was treated with MOS solution for 1 week after anastomosis surgery. An area in the square (**a**) corresponds to an area in the square (**a**) in **Figure 4**. ***B.*** A microscopic image of a longitudinal section, prepared following fixation, that was taken along the line (**b**) indicated in panel ***A***.

A stereomicroscopic image demonstrating the thick granulation tissue at the anastomotic region in mouse treated with MOS solution for 1 week after surgical anastomosis is shown in **Figure 3** ***A***. However, under a stereomicroscopy no nerve cells or fibers were visible. A longitudinal section including the same granulation tissue indicates an obvious thickness of the tissue (**Figure 3** ***B***).

**Figure 4:**
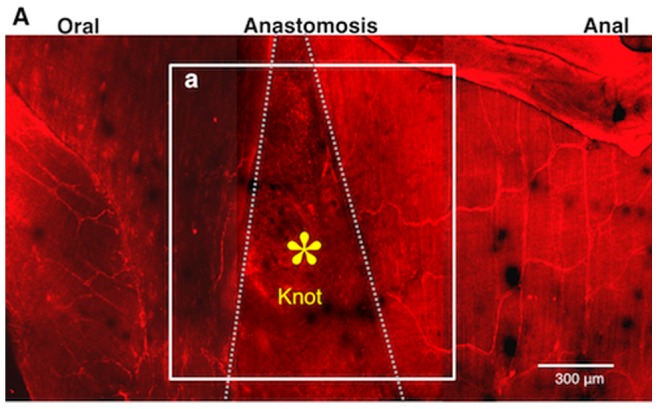
Immunohistochemical image for anti-neurofilament (NF) antibody of a whole mount preparation of the same intestine shown in **Figure 5**. *A–a* corresponds to Figure 5*A* (the image by 2PM). *, A knot of thread in the area between two-dotted lines indicates the anastomotic area. The granulation tissue was removed to allow for laser penetration. Normal myenteric plexus in the intact oral and anal sites are visible, but nerve cells and fibers are not visible in the anastomotic region because of the thickness of the anastomotic area.

**Figure 5:**
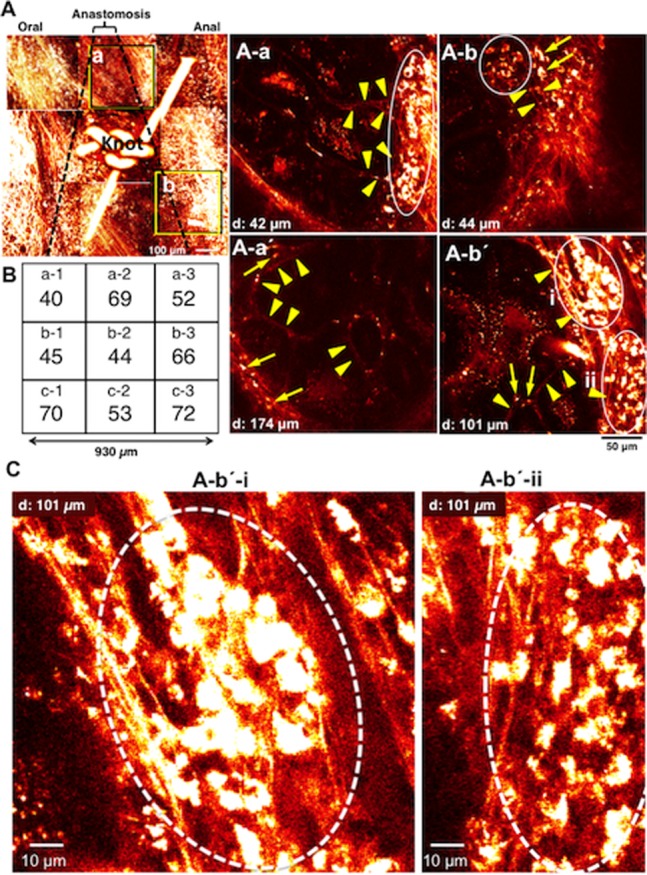
Images of anastomotic region of the terminal ileum in a MOS-treated mouse. The dotted lines indicates the anastomosis site. Around the knot of thread we obtained each image from 9 visual fields. ***A.*** Images stacked with Z axis to a total depth of 200–300 µm. ***A–a.*** image 42 µm deep to the serosa surface in area (***a***) in ***A***. ***A–a’.*** image 174 µm deep to the serosa surface in the same area (***a***) in ***A***. ***A–b.*** 44 µm deep to the serosa surface in area (***b***) in ***A***. ***A–b’.*** image 101 µm deep to the serosa surface in the same area (***b***) in ***A***. Arrows indicate nerve cells in ***A–a’***, ***b*** and ***b’***, and arrowheads indicate nerve fibers in ***A–a***, ***a’***, ***b*** and ***b’***, and circles indicate ganglion-like clusters of neurons in ***A–a***, ***b*** and ***b’***, respectively. ***B.*** Number of neurons in each field (size: 310 µm×310 µm) around the knot. ***C.*** Newborn nerve cells formed ganglion structures indicated by circles. These were enlarged from the images shown in ***A–b’–i*** and ***–ii***.

Using confocal imaging of the fixed whole mount preparation, no nerve cells or fibers were visible in the granulation tissue at the anastomosis, although intact myenteric plexus was visible in the intact area in a mouse treated with MOS solution for 1 week after surgery (**Figure 4**). The living ileum before fixation is shown in Figure 5.

In mice treated with MOS solution for 1 week after anastomosis, new ganglia-like structures and nerve fibers were observed by *in vivo* imaging of the thick granulation tissue at the anastomotic region of the living ileum (**Figure 5** ***A***). In sites both oral (**Figure 5** ***A*** ***–a*** **&** ***-a’***) and anal (**Figure 5** ***A*** ***–b*** **&** ***−b’***) to the anastomotic site, new ganglia-like structures packed with many of newborn neurons and interganglionic nerve fibers were apparent in each image regardless of the depth from the serosa indicating that in the granulation tissue a new enteric neural network was being formed. The density of neurons observed within the anastomosis was 511 cells per 864,900 µm^2^. The distribution of neurons was even in each nine field (from a−1 to c−3) (**Figure *5B***). **Figure 5** ***C*** clearly illustrates new neurons in two typical ganglion-like structures.

**Figure 6:**
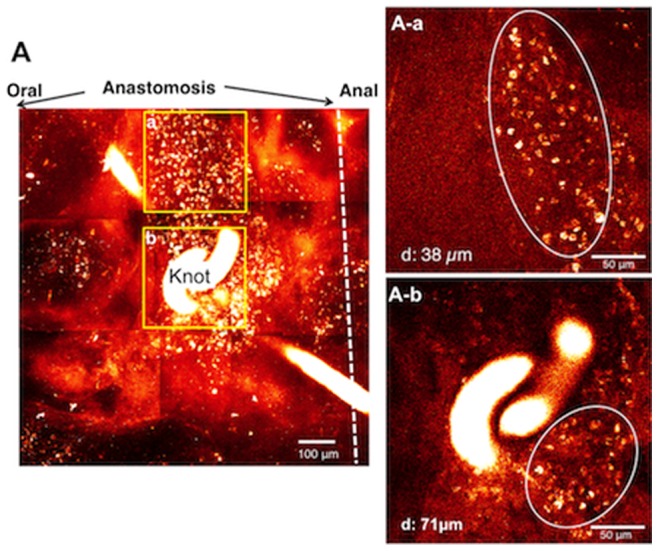
Images of anastomosis of the ileum in an SB-207266 (SB) plus MOS treated mouse. SB plus MOS treatment was performed for one week. ***A.*** Images stacked in the Z axis with a total depth of 200 - 300 µm. ***A–a.*** image 38 µm deep to the serosa surface in area (***a***) in ***A***. ***A–b***. image 71 µm deep to the serosa surface in area (***b***) in ***A***. Circles indicate aggregates of small non-neuronal cells (***A–a*** and ***b***), respectively.

In mice treated with the 5-HT_4_ antagonist, SB 207266 (SB) plus MOS solution for 1 week after anastomosis, no neurons or nerve fibers were observed in the anastomotic region (**Figure 6**), although aggregates of small cells (not neurons) were observed near the surface (**Figure 6** ***A*** ***–a*** **&** ***–b***). Thus, enteric neurogenesis was largely suppressed by simultaneous administration of the 5-HT_4_ receptor blocker, SB along with MOS. Similar results were obtained with all 4 mice treated with MOS and SB.

Using confocal imaging of fixed, whole mount preparations, no nerve cells or fibers were visible in the granulation tissue at the anastomosis, although intact myenteric plexus was visible in the intact area in a mouse treated with SB and MOS solution for 1 week after surgery (data not shown).

**Figure 7:**
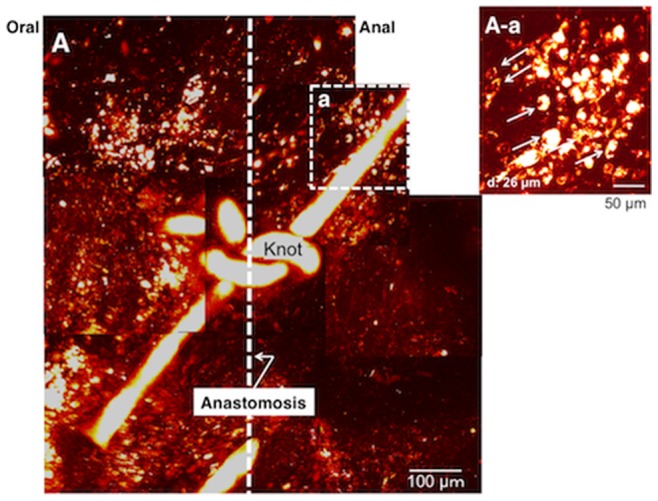
Images of around the suture knot at anastomosis of the ileum in DMSO treated mouse. Vehicle treatments were performed for 4 weeks. The dotted line indicates the anastomosis. ***A.*** Images stacked with Z axis up to a total depth of 151 - 201 µm. ***A–a.*** image 26 µm deep to the serosa surface close to the thread around the knot. A small number of neurons are visible.

Vehicle treated mice underwent in vivo imaging of the anastomotic region at 1 week (n = 5) and 4 weeks (n = 4) after ileum transection and re-anastomosis (**Figure 7**). One week after surgery, neither nerve bundles nor ganglia were visualized at the anastomosis. In contrast, 4 weeks after surgery, a small number of neurons were detected in one preparation (**Figure 7** ***A*** ***–a***). In the other three mice treated with vehicle for 4 weeks after surgery, no neurons were detected at any depth within the granulation tissue.

**Figure 8:**
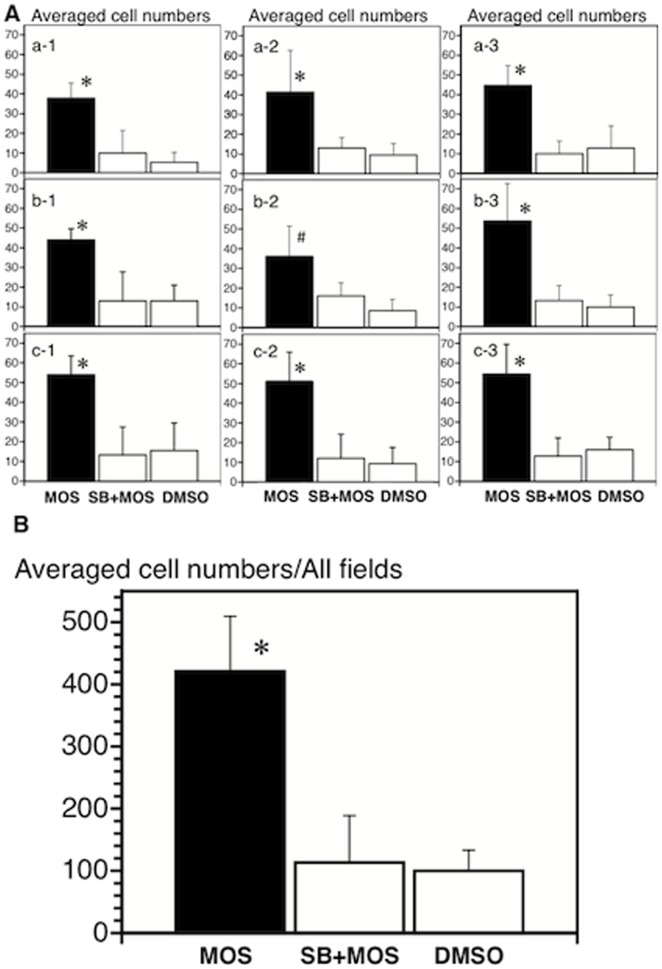
The average cell numbers in each of nine fields (A) and all fields (B) at the anastomosis. The comparison was performed among MOS (n = 5), SB+MOS (n = 4) and vehicle-treated (n = 4) mice. Each of the nine fields corresponds to that in Figure 5B. *, P<0.05 vs. SB+MOS and vehicle. #, P<0.05 vs. vehicle.

The average number of neurons observed amongst nine fields within the anastomosis in mice treated with MOS solution was significantly (P<0.05) larger than that in SB plus MOS treated mice (n = 4) or DMSO-treated mice (n = 4) after anastomosis (**Figure 8** ***A***). New neurons were observed without oral or anal and mesenteric or anti-mesenteric localizations in any of the three groups (**Figure 8** ***A***).

The average density of neurons observed in all fields within the anastomosis in mice treated with MOS solution was 421±89 per 864,900 µm^2^ (n = 5), significantly (P<0.05) higher than SB plus MOS treated mice (113±76 per 864,900 µm^2^; n = 4) or mice treated with vehicle (100±34 per 864,900 µm^2^; n = 4) (Figure 8B). Moreover, the average number of neurons distributed at the anastomosis in MOS treated mice was about 5 cells per 10,000 µm^2^, compared to 35 cells per 10,000 µm^2^ (ganglia areas) in the intact small intestine of mice [11].

**Figure 9:**
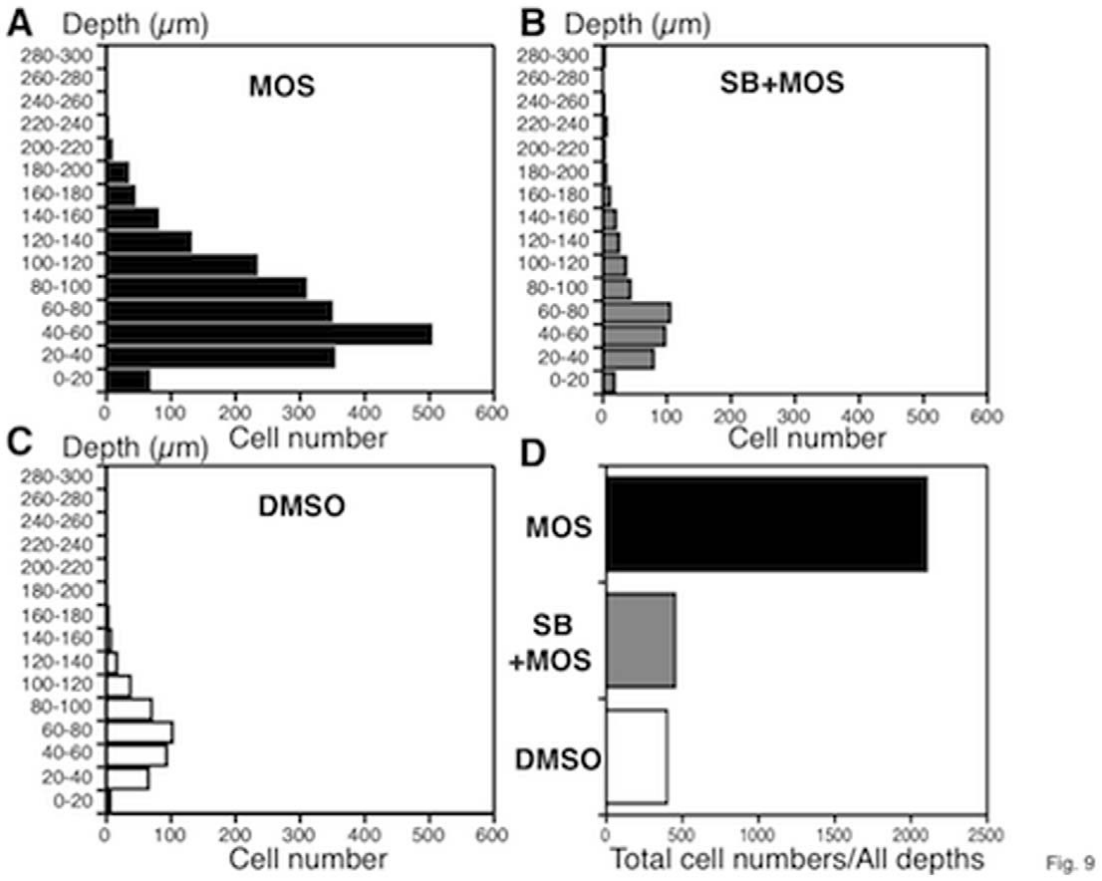
The distribution of total neurons in MOS (n = 5), SB+MOS (n = 4) and vehicle-treated (n = 4) mice. *A, B, C.* Number of total neurons at depths of every 20 µm. *D.* Cumulative numbers from all depths.

The distribution of neurons in depth was analyzed at depths of every 20 µm. In all three groups almost all neurons were located within 100 µm of the surface (**Figure 9** ***A–C***). The total number of neurons in MOS-treated mice was about four-fold of that in SB plus MOS and DMSO treated mice (**Figure 9** ***D***).

**Figure 10:**
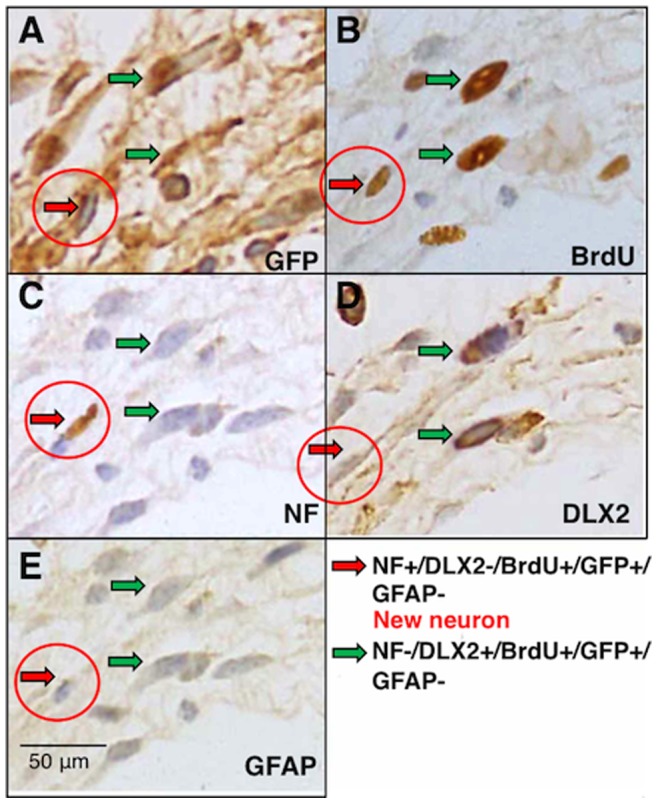
Correctly identified fluorescent neurons by 2PM are proved to be neurons at the anastomosis in MOS-treated mice. *A.* Green Fluorescent Protein (GFP)-positive cells. *B.* 5-bromo-2'-deoxyuridine (BrdU)-positive cells. *C.* A neural marker, neurofilament (NF)-positive cell. *D.* A neural stem cell marker, distal less homeobox 2 (DLX2)-positive cells. *E.* glial fibrillary acidic protein (GFAP)-negative cells. Red arrows indicate NF+/DLX2−/BrdU+/GFP+/GFAP- cell: this cell is a new neuron. Green arrows indicate NF−/DLX2+/BrdU+/GFP+/GFAP- cells: these cells seem to be neural progenitors. Similar results are obtained in other preparations.

Correctly identified fluorescent neurons by 2PM are proved to be neurons with an independent technique at the anastomotic site. NF-positive, DLX2-negative, BrdU-positive and GFP-positive cell is identified as a new neuron (**Figure 10** ***A–D***). NF-negative, DLX2-positive, BrdU-positive and GFP-positive cells seem to be neural progenitors. At this anastomotic site, GFAP-positive enteric glial cells are not found (**Figure 10** ***E***).

## Discussion

This is the first study involving in vivo imaging of enteric neurons with 2PM, although in vivo imaging of enteric neurons with confocal laser endomicroscopy has been recently performed [9]. We detected the formation of newly generated neurons in the thick granulation tissue at the site of anastomosis. Imaging with 2 PM allowed enteric neural imaging several hundred microns deep within the gut of living mouse. In contrast to the brain tissue [7], the structure of the gut tissue is complex, consisting of multiple layers and tissue types, including mucosa, submucosa, circular and longitudinal muscles, blood vessels and crypt glands. Therefore, to enhance visualization of enteric neurons we used Thy1-GFP mice [8] after confirmation of expression of cytoplasmic GFP in enteric neurons in preliminary studies. In the present study, newly formed enteric neurons also expressed cytoplasmic GFP. In future studies, we are planning functional studies of enteric neurons using in vivo imaging with 2PM and genetically encoding calcium indicators [12].

A critical obstacle to overcome in order to obtain clear images of enteric neurons in vivo was to suppress motion disturbance associated with gut motility. Otherwise, observed images would be blurry and non-interpretable. We found that pinning and intraluminal injection of papaverine eliminated tissue movement and allowed for the acquisition of sharp images.

One week after surgical anastomosis, MOS facilitated formation of newly generated enteric neurons in the granulation tissue at the anastomosis. However, even 4 weeks after surgery, only a small number of newborn neurons were identified in the granulation tissue of vehicle-treated control animals. The effects of MOS on neurogenesis were completely antagonized by treatment with a 5-HT_4_ receptor antagonist, indicating that MOS facilitated formation of newborn enteric neurons via 5-HT_4_-receptor activation. Although the number of newly formed enteric neurons was significantly higher in the MOS-treated mice as compared to antagonist treated and vehicle controls, the distribution pattern of newly formed enteric neurons was similar, i.e., neurons were distributed close to the edge of the granulation tissue. This suggested the possibility that neural stem cells were mobilized from the outside of the granulation tissue.

Enteric nervous system (ENS) development is relevant to Hirschsprung’s disease (HSCR; congenital aganglionosis of the terminal bowel) and related diseases, which are still imperfectly treated. It is well known that mutations in genes encoding the Ret receptor tyrosine kinase and endothelin receptor type B are involved in HSCR pathogenesis [13], [14]. We found MOS increased mRNA of c-Ret receptor tyrosine kinase in a rat model and that a 5-HT_4_ receptor antagonist completely blocked this effect [15]. Therefore, it seems likely that the target molecule of MOS is the Ret receptor tyrosine kinase.

Enteric neurogenesis must be strictly controlled, because hyperplasia of enteric neurons due to hypersensitivity for glial cell-derived neurotrophic factor (GDNF)-Ret signaling reversely results in HSCR [16]. Nevertheless, treatment with 5-HT_4_ receptor agonists such as MOS could be a promising tool to treat HSCR and related disorders.

In conclusion, *in vivo* imaging by 2PM allowed for high-resolution deep imaging of the intestines *in vivo*. Thick granulation tissue at the site of anastomosis, including newly formed ganglion-like structures and nerve fibers, could be studied in the intact murine small intestine, whereas this would have been impossible with traditional fluorescence or confocal microscopy. The results presented here confirmed that oral administration of MOS promotes the generation of enteric neurons by activation of enteric neural 5-HT_4_-receptors in the murine small intestine. The present technology would be promising for *in vivo* imaging of enteric neurons distributed throughout the entire gastrointestinal tract as a means of evaluating enteric neural function and dysfunction in the normal gut and in, for example, diabetic [17] and parkinsonism mouse models [18].

The recent publications suggest that mouse enteric glia can be neuronal precursors and thus form neurons *in vitro* and *in vivo* under specific circumstances [19]–[21]. Therefore, we have investigated glia and/or their relation to the newly formed “neurons”. However, we did not found any enteric glial cells at the anastomotic site. It seems unlikely that enteric glial cells contribute to neurogenesis at least at the anastomotic site.
